# Duplication of subtelomeric regions in an adult with acute monocytic leukemia with an acquired jumping translocation involving 3q13.31-qter

**DOI:** 10.1016/j.dib.2017.06.043

**Published:** 2017-07-04

**Authors:** Eigil Kjeldsen

**Affiliations:** Cancercytogenetics Section, Hemodiagnostic Laboratory, Department of Haematology, Cancer and Inflammation Center, Aarhus University Hospital, DK-8000 Aarhus C, Denmark

## Abstract

A jumping translocation (JT) involves a single donor chromosome and two or more recipient chromosomes in which a similar chromosomal region is translocated to various recipient chromosomes in different cell lines of a single individual. JTs are often associated with telomeric regions. Only 21 acquired JTs have previously been described in myeloid malignancies. Three of these cases involved the 3q13.31-qter region of which all were associated with a dismal outcome. In our recent publication, “Characterization of an acquired jumping translocation involving 3q13.31-qter in a patient with *de novo* acute monocytic leukemia” [1], we characterized the breakpoint region 3q13.31 by oligo-based array comparative genomic hybridization analysis. The present article provides data on copy number aberrations observed in the subtelomeric regions of this patient. Copy number alterations in the subtelomeric region have not been addressed previously in patients with JT.

**Specifications Table**

**Table:** 

Subject area	*Biology*
More specific subject area	*Cancer genomics*
Type of data	*Table and Figure*
How data was acquired	*Oligo-based array-comparative genomic hybridization (oaCGH) was used to examine for copy number alterations*
Data format	*Analyzed*
Experimental factors	*Purified DNA from bone marrow samples at the time of acute monocytic leukemia diagnosis and at the time of complete remission were compared*
Experimental features	*Direct comparison by oaCGH analysis using purified DNA from time of complete remission as reference DNA allows to distinguishing whether observed copy number alterations are true copy number alterations or normal copy number variations*
Data source location	*Aarhus, Denmark*
Data accessibility	*Data is with this article*

**Value of the data**

• Copy number alterations in subtelomeric regions examined by oaCGH analysis are rarely reported in leukemic genomes
• Copy number variations are common in subtelomeric regions when foreign DNA is used as reference DNA making it impossible to distinguish these from true copy number alterations
• Direct comparison of patient׳s leukemic DNA with purified DNA from the time of complete remission allows for reporting of all copy number aberrations fulfilling basic criteria for them to be called
• The findings add to the spectrum of subtelomeric copy number alterations associated with JT

## 1 Data

**Fig. 1:**
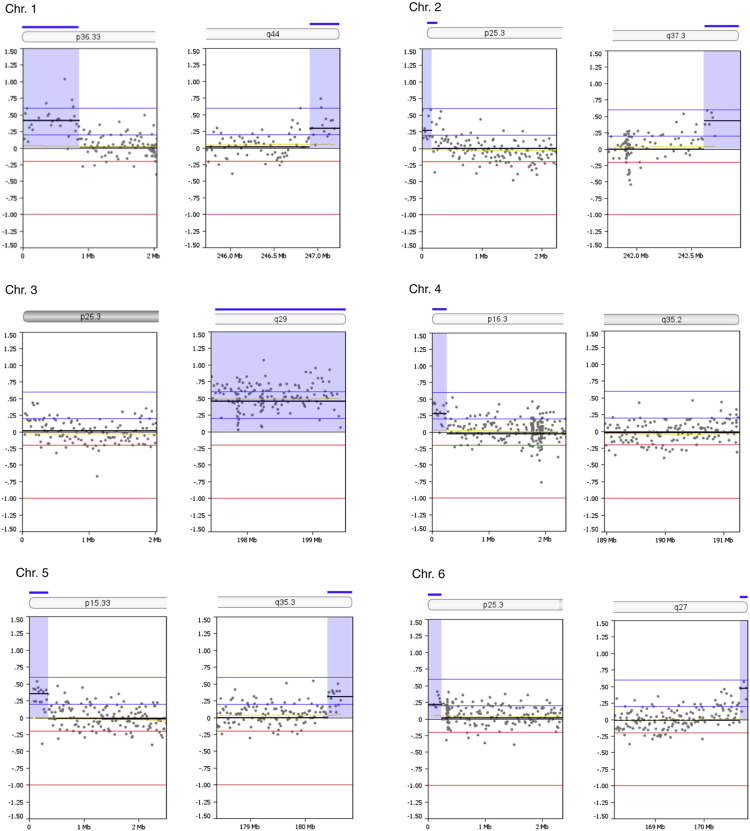

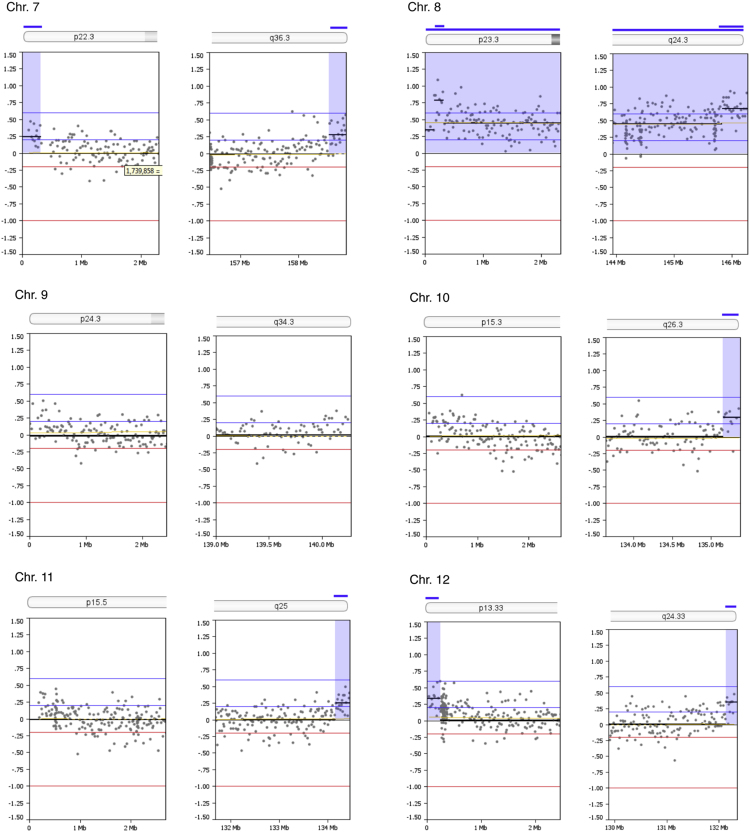

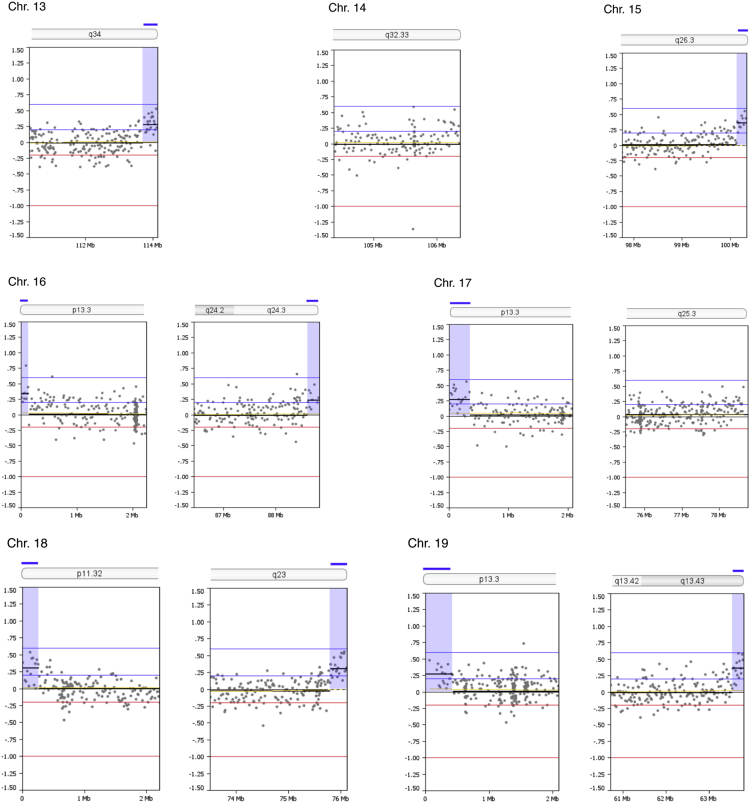

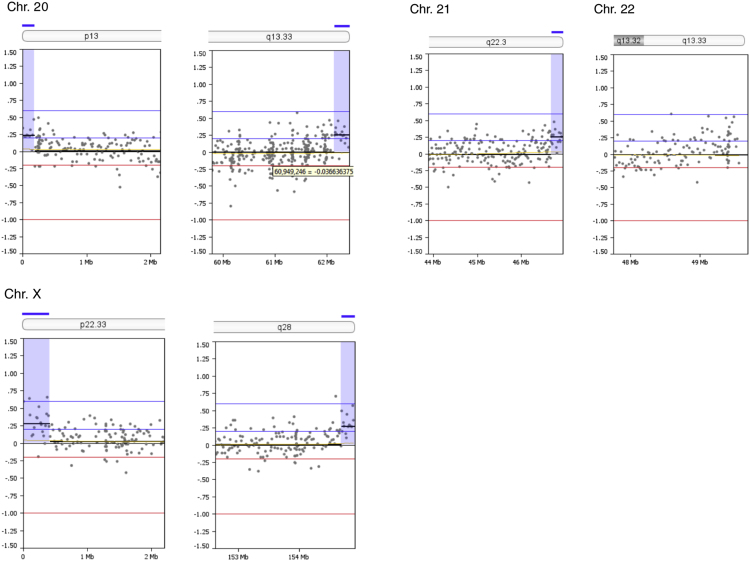
Zoom-in view of telomeric p- and q-regions of individual chromosomes 1-22 and the X-chromosome of oaCGH analysis using DNA from diagnosis and DNA from complete remission as reference. The x-axis indicates genomic positions and the y-axis indicates the log2 ratios. Blue shades indicate gained regions.

Copy number alterations at subtelomeric regions were examined. Thirty one out of 92 subtelomeric regions (33.7%) had duplications between 141,682 and 864,400 bp in size (mean size 298,957 bp) as summarized in Table 1. The genomic profiles of all subtelomeric chromosomal regions are shown in Fig. 1. A search in the UCSC bioinformatics database revealed a total of 177 genes in the subtelomeric regions with segmental duplications (Table 2).

**Table 1:** Subtelomeric copy number alterations in the blast cells.

Chromosome	Cytoband	Genomic position (bp)^a^	Size (Mb)	Copy number aberration
1	p36.33 q44	1 - 864,399 246,909,654 – 247,249,719	0.86 0.34	Gain Gain
2	p25.3 q37.3	1 - 168,438 242,618,961 – 242,951,149	0.17 0.33	Gain Gain
4	p16.3	1 - 259,496	0.26	Gain
5	p15.33 q35.3	1- 350,106 180,403,896 – 180,857,866	0.35 0.45	Gain Gain
6	p25.3 q27	1 - 240,983 170,743,310 – 170,899,992	0.24 0.16	Gain Gain
7	p22.3 q36.3	1 - 317,221 158,527,361 – 158,821,424	0.32 0.29	Gain Gain
8	p23.3 q24.3	1 - 158,984 145,836,948 – 146,274,826	0.16 0.44	Gain Gain
10	q26.3	135,156,343 – 135,374,737	0.22	Gain
11	q25	134,158,206 – 134,452,384	0.29	Gain
12	p13.33 q24.33	1 - 248,197 132,134,110 – 132,349,534	0.25 0.22	Gain Gain
13	q34	113,707,862 – 114,142,980	0.44	Gain
15	q26.3	100,135,399 – 100,338,915	0.20	Gain
16	p13.3 q24.3	1 - 141,681 88,600,659 – 88,827,254	0.14 0.23	Gain Gain
17	p13.3	1 - 353,878	0.35	Gain
18	p11.32 q23	1 - 266,608 75,796,416 – 76,117,153	0.27 0.32	Gain Gain
19	p13.3 q13.43	1 - 430,031 63,549,249 – 63,811,651	0.43 0.26	Gain Gain
20	p13 q13.33	1-185,691 62,142,263 – 62,435,964	0.19 0.29	Gain Gain
21	q22.3	46,677,243 – 46,944,323	0.27	Gain
X	p22.33 q28	1 - 412,524 154,690,971 – 154,913,754	0.41 0.22	Gain Gain

a Genomic positions are given according to NCBI build 36.1 (hg18).

**Table 2:** Genes located in the subtelomeric regions within identified segmental duplications.

Chromosome	UCSC RefSeq Genes

1	1p: *DOX11L1, WASH7P, MIR6859, MIR6723, FAM87B, FAM138A, LINC00115, LINC01128, ORF4, ORF29* 1q: *SCCPHD, LINC01341, AHCTF1, ZNF670-695, ZNF670*
2	2p: *FAM110C* 2q: *DTYMK, ING5, D2HGDH, GAL3ST2, PABL, NEU4, PDCD1, CXXC11*
4	4p: *ZNF595, ZNF718, ZNF876P*
5	5p: *PLEKHG4B, LRRC14B, CCDC127, SDHA, HRAT5, PDCD6, AHRR* 5q: *CNO6, SCG3A1, FLT4, ORY1, MGAT1, HEIH, ZFP62*
6	6p: *LINC00266-3* 6q: *PSMB1, TBP, PDCD2*
7	7p: *LOC10723672, LOC100507642, LOC105375115, FAM20C* 7q: *ESYT2, WDR60, LINC00689, VIPR*
8	8p: *OR4F21* 8q: *ARHGAP39, ZNF251, RPL8, ZNF34, ZNF7, MIR6850, COMMD5, ZNF250, ZNF16, ZNF252P, TMED10P1, ZNF252P-AS*
10	10q: *PRAP1, FUOM, MIR3944, ECHS1, PAOX, MTG1, SPRN, SCART1, CYP2E1, SYCE1*
11	11q: *GLB1L3, GLB1L2, B3GAT1, LOC283177*
12	12p: *FAM138D, DDX11L1, LOC100288778, IQSEC. 3* 12q: *SFSWAP, MMP17*
13	13q: *MCF2L, F7, F10, PROZ, PCID2, CUL4A, MIR8075, LAMP1, GRTP1, ADPRHL1, LOC101928841, DCUN1D2*
15	15q: *MEF2A, LYSMD4*
16	16p: *DDX11L10, MIR6859, WASIR2, POLR3K, SNRNP25, RHBDF1, MPG, NPRL3* 16q: *ZC3H18, IL17C, CYBA, SNAI3, MVD, RNF166, CTU2, MIR4722, PIEZO1, LOC100289580*
17	17p: *DOC2B, RPH3AL, LINC02091, LOC100506388, LOC105371430, C17orf97, RFLNB*
18	18p: *DUX4, LOC102723376, MIR8078, ROCK1P1, USP14, THOC1* 18q: None
19	19p: *WASH5P, MIR1302, FAM138A, FAM138F, OR4F17, LINC01002, PLPP2, MIER2, THEG, C2CD4C, SHC2* 19q: *ZNF8, ZBTB45, A1BG, MZF1*
20	20p: *DEFB125, DEFB126, DEFB127, DEFB128* 20q: *PPDPF, PTK6, FNDC11, HELZ2, GMEB2, MHENCR, STMN3, RTEL1, ARFRP1, TNFRSF6B, ZGPAT, LIME1, SLC2A4RG, ZBTB46*
21	21q: *LINC00334, POFUT2, LINC00316, COL18A1, MIR6815, SLC19A1*
X	Xp: *PLCXD1, GTPBP6, LINC00685, PPP2R3B* Xq: *TMLHE, LOC101927830, SPRY3*

## 2 Experimental design, materials and methods

By oaCGH analysis using purified DNA from aspirated bone marrow at the time of diagnosis and purified DNA from aspirated bone marrow at the time of complete remission as reference DNA we recently characterized the 3q13.31 breakpoint region in an adult with *de novo* acute monocytic leukemia harboring an acquired JT involving the 3q13.31-qter chromosomal region [1]. This experimental design allows for a direct comparison between the patient׳s bone marrow cells at diagnosis and at complete remission eliminating interpretations of possible copy number variations, which are known to be common in subtelomeric regions [2]. Such a direct comparison allows for reporting of all copy number aberrations, which fulfills the basic criteria for them to be called. Reference genome was NCBI build 37 (hg19) and the University of California Santa Cruz (UCSC) database (http://genome.ucsc.edu) was used for bioinformatics analysis.
